# Regional COVID-19 Dynamics: Surrogate Synchrony in Case Infection Rates

**DOI:** 10.3389/fpubh.2021.647441

**Published:** 2021-08-26

**Authors:** Samantha Robinson

**Affiliations:** Department of Mathematical Sciences, University of Arkansas, Fayetteville, AR, United States

**Keywords:** COVID-19, dyadic processes, dynamic systems, synchrony, disease surveillance, disease prevention, regional dynamics

## Abstract

As many jurisdictions consider in-person learning strategies (including at Institutions of Higher Education, IHE), implementing travel restrictions or quarantines, and/or establishing interstate pacts to reduce COVID-19 spread, this study explores the degree to which COVID-19 case infection rates in a group of neighboring, Southern and Midwestern U.S. states (namely, Arkansas and its contiguous neighbors) are patterned in a non-random way known as synchrony. Utilizing surrogate synchrony (SUSY) to estimate the dyadic coupling between the COVID-19 case infection rate processes in this region from March to December 2020, results indicate that significant synchrony is present between Arkansas and three of its neighbors. The highest level of instantaneous synchrony occurs between Arkansas and Tennessee, with the next highest level occurring between Arkansas and Missouri. There is evidence of directionality in the synchrony, indicating that Arkansas case infection rates lead Mississippi while rates in Missouri and Tennessee lead Arkansas. The lagged cross-correlations suggest the greatest synchrony to occur between 3 and 6 days. To explore the effect of IHE reopening on COVID-19, synchrony is compared between pre- and post-reopening windows. Results suggested that, following reopening, there are gains in detectable synchrony and that COVID-19 is in-flowing to Arkansas from all of its neighboring states. Taken together, results suggest that there is spatiality to COVID-19 with neighboring states having case infection rates that are significantly synchronous at a lag time that would be expected based on symptom onset. This synchrony is potentially strengthened by the in-flow and cross-border movement of IHE students.

## Introduction

Severe Acute Respiratory Syndrome Coronavirus 2 (SARS-CoV-2), the virus that causes the Coronavirus Disease 2019 (COVID-19), was originally identified in the Chinese city of Wuhan, the capital city of the Hubei province, in December 2019. The Chinese government, in an effort to limit the spread of COVID-19, implemented large-scale social distancing measures, the strictest of which was the complete lockdown of Wuhan (1). Such measures were applauded by the World Health Organization (WHO) and models have demonstrated that tightly controlling the movement of people substantially reduces virus spread (1–3).

Despite Chinese lockdown measures and mitigation strategies, COVID-19 emerged all around the world and, by early 2020, was rapidly spreading throughout Europe and parts of the United States. By March the WHO had declared COVID-19 a pandemic (4). Additional lockdowns and stay-at-home orders of various forms were implemented across the world. California would be the first U.S. state to issue a mandatory stay-at-home order and, eventually, all but five U.S. states would issue some type of advisory order, shuttering businesses, and keeping schools closed to in-person instruction for the remainder of the spring (5).

By late April, attempts were already being made across the United States to strategically reopen (6). Many states drafted phased reopening plans with gating criteria while some states even participated in multi-state regional approaches, such as the Midwest Partnership, the Northeast Multi-State Council, and the Western States Pact (6). This regional approach to reopening is supported by preliminary evidence that COVID-19 disease transmission might have a spatiality that arises from local economic structures (7).

Van Pelt et al. noted that the pandemic forced Institutions of Higher Education (IHE) in the United States (i.e., institutions engaging in post-secondary, tertiary education) to transition to virtual instruction quickly during spring 2020, disrupting the operations of over 4,000 IHE and impacting the education of more than 25 million students (8). While much of the initial planning for reopening in April and May 2020 was focused on reopening specific sectors of the economy, primary and secondary schools as well as IHE also began to draft plans for fall instruction.

The College Crisis Initiative (C2i), an initiative of Davidson College, began surveying IHE to understand fall semester plans. Of approximately 3,000 IHE (including both public and private 2-year and 4-year institutions), only 10% committed to fully online instruction for the fall 2020 term (9). The remaining 90% of institutions, even if planning for fall instruction to be delivered primarily online, maintained that there would be some in-person coursework offered. With that promise of in-person, face-to-face instruction in IHE, cross-state movement of younger individuals would no longer be as tightly controlled as it had been during the spring and summer months. This increase in necessary cross-state movement of college students, a high proportion of which are likely to be asymptomatic (10, 11), had the potential to increase the spread of COVID-19. In fact, infections did spike and the virus death rate rose faster than the national average in counties across the United States where college students make up 10% or more of the population (12, 13).

The purpose of this study was to explore the regional dynamics of COVID-19 infections between a group of neighboring states in the Southern and Midwestern regions of the United States. Specifically, this study investigated the synchrony between Arkansas COVID-19 infections with that of its contiguous neighbors, while making particular note of any differences in synchrony observed before and after the August reopening of IHE in the region. Case infection rates of each state that are patterned or synchronized to a significant degree would suggest that the interacting processes driving COVID-19 in these neighboring states are or have become correlated at a level exceeding random chance (14). Detection of such synchrony could help inform higher education stakeholders, along with public health officials, about the case infection dynamics of a particular region and, thus, guide the development of recommendations for instructional plans at IHE during the pandemic.

## Method

### Regional Selection and Data Source

Arkansas has six contiguous neighbors: Louisiana, Mississippi, Missouri, Oklahoma, Tennessee, and Texas. While the state was one of only five U.S. states to never issue a stay-at-home order, many businesses and schools (including IHE) closed temporarily in the spring.

According to the most recent Rural Profile of Arkansas (15), Arkansas is a very rural state. When using the Office of Management and Budget Metropolitan Statistical Area county-based definition of rural, 41% of Arkansans live in rural counties as of 2017 compared to only 14% of the U.S. population as a whole. Despite the rural nature of the state, White House coronavirus task force reports leaked to the Center of Public Integrity reveal that Arkansas has been in the red zone since at least June in terms of cases and in the red zone for deaths since at least August (16–21). The virus spread is unyielding in Arkansas, which is currently (as of December 2020) in the red zone for cases, test positivity, deaths, and hospital admissions (21).

Arkansas has a number of IHE, a majority of which planned to reopen either in a hybrid or fully in-person manner for the fall 2020 term (9). Similar to the larger national picture of IHE fall instructional plans, approximately 10% of all 4-year IHE in the state planned to be fully online. However, that 10% represents only 0.57% of the 4-year IHE student enrollment in the state (9). At the largest IHE in the state, 46% of all enrolled students are from out of state and nearly 35% of all enrolled students come from contiguous neighbors of Arkansas (22).

Since one interest of the current study was to explore regional dynamics of COVID-19 infections with particular attention paid to cross-state travel following IHE reopening, Arkansas was selected given a unique combination of characteristics, namely,

- Arkansas has six contiguous neighbors, making it tied for the fifth most connected U.S. state.
- Two of six contiguous neighbors of Arkansas, Missouri and Tennessee, tie for having the most shared borders of any other states in the United States.
- Arkansas is a very rural state and, hypothetically, should have been shielded from virus transmission and spread in the early weeks and months of the pandemic.
- Following IHE reopening, Arkansas has continued to be listed in the red zone according to White House coronavirus task force reports and is in a critical stage with unyielding community transmission.
- More than 99% of students enrolled at 4-year IHE in the state were offered some in-person learning opportunities for fall 2020 and many of these students are from out of state.

Time series data of COVID-19 case infection rate 7-day moving averages were obtained on December 19, 2020, from the Centers for Disease Control and Prevention (CDC) COVID-19 Case Surveillance Public Use Data (23). According to the CDC, rates are calculated using U.S. Census Bureau, 2018, American Community Survey 1-year estimates and are shown as cases per 100,000 people, with the 7-day moving average of new cases calculated to smooth expected variability in daily counts. Time series used in the current study began on March 24 and ended on December 17, 2020. The start of each series was determined by selecting the first date on which each of all seven states had started reporting confirmed COVID-19 infections. The end of the series was selected to minimize the effect of an explainable December 18 outlier, as Texas began reporting probable cases on this day, resulting in a total of 184,758 cases reported in 1 day (23). It should be noted that all data used in this study is considered provisional according to the CDC (23).

All ethical standards of data collection were adhered to during this study. The COVID-19 Case Surveillance Public Use Data is publicly available and accessible through the CDC COVID Data Tracker (*c.f*. https://covid.cdc.gov/covid-data-tracker).

### Analytic Approach

All data pre-processing and analyses were performed in R (24).

Synchrony is roughly defined as the degree to which two interacting processes or systems are patterned or synchronized in timing and in the form in a non-random way (14, 25). Instantaneous synchrony would suggest that the systems are correlated beyond random chance with no lag time. However, synchrony can occur with a dynamic lag such that there is a direction of entrainment whereby one system entrains the other.

In order to detect synchrony that may not be instantaneous and to determine the direction of entrainment of the COVID-19 cases in these neighboring states, an algorithm based on time-lagged cross-correlations of bivariate time series was utilized in the current study, that is, the surrogate synchrony (SUSY, *c.f*. www.embodiment.ch) algorithm (26).

The bivariate time series is split into segments of a set size, with cross-correlations computed segment-wise at all time lags up to a selected maximum lag time. The lagged cross-correlations are transformed using Fisher's *Z* transformation to allow for aggregation, with a segment-wise synchrony represented by the average of all transformed, lagged cross-correlations within each segment. The segment synchronies are then aggregated with an overall synchrony determined by averaging over all segment-wise synchrony measures in the entire time period. Typically, absolute values of the lagged cross-correlations are utilized to detect “anti-phase” and “in-phase” synchrony, that is, consistently negative or positive associations. However, in order to differentiate between in-phase and anti-phase coupling, SUSY can be conducted without absolute lagged cross-correlation values.

Effect sizes and statistical significance can be assessed utilizing permutation. Randomized segment shuffling creates a permuted sampling distribution of the overall synchrony measure, which can then be utilized to determine effect sizes and statistical significance for synchrony measured with or without absolute values as well as for the direction of entrainment.

In the current study, the segment size for the dyadic time series was set to be 28 days with a maximum lag time of 7 days. Both absolute and non-absolute lagged cross-correlations were evaluated, and the direction of entrainment was of interest.

SUSY was implemented for the COVID-19 case infection rate 7-day moving averages between Arkansas and each of its contiguous neighbors for the entire time period from March to December as well as separately for each distinct pre- and post-IHE reopening time period.

## Results

### Descriptive

Each state-level time series consisted of 269 days of provisional data on COVID-19 case and case infection rate 7-day moving averages. The pre- and post-IHE reopening time series consisted of ~130 and 139 days, respectively, from March to July and from August to December.

**Figure 1:**
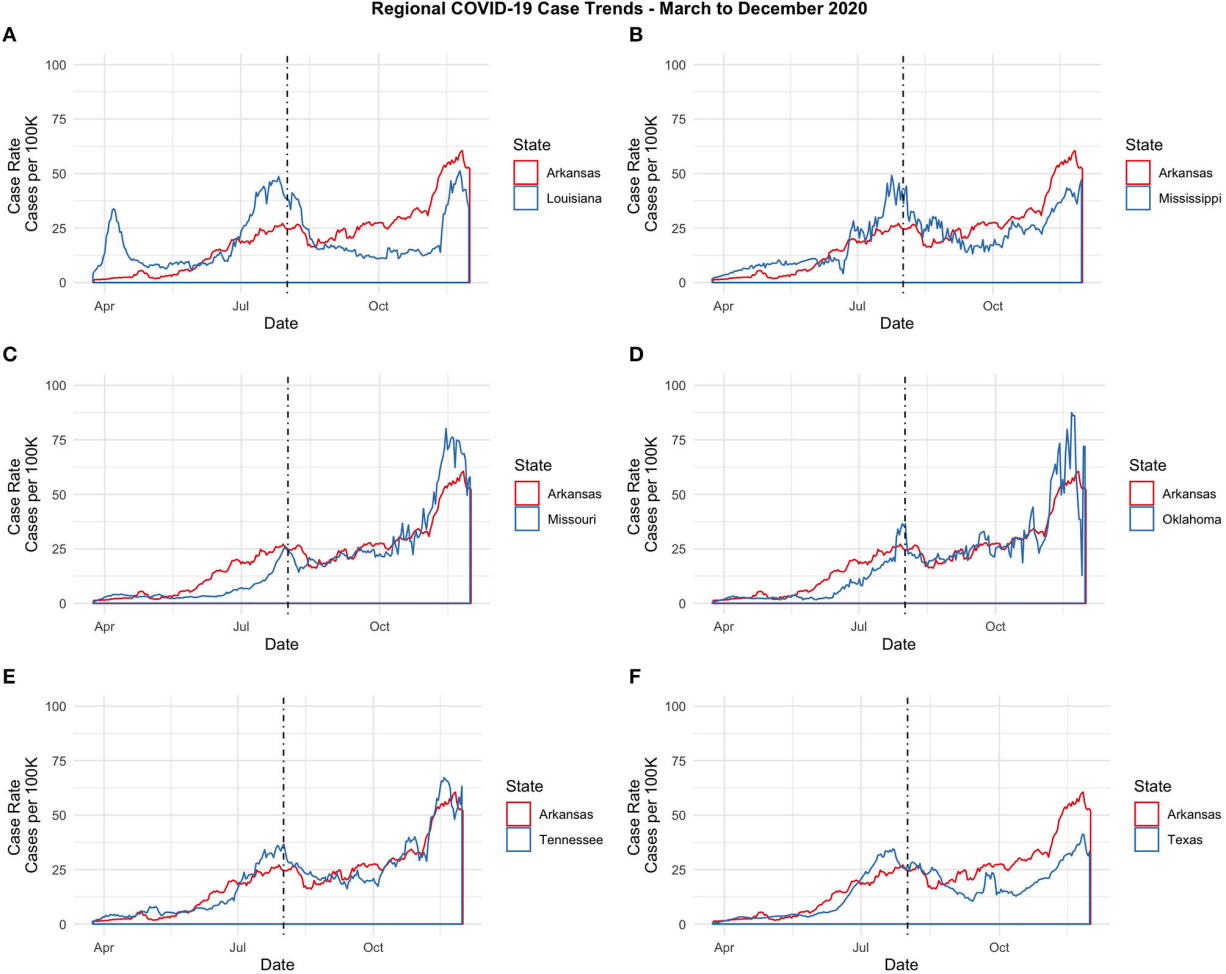
Regional COVID-19 Case Trends-March to December 2020. **(A)** Arkansas and Louisiana. **(B)** Arkansas and Mississippi. **(C)** Arkansas and Missouri. **(D)** Arkansas and Oklahoma. **(E)** Arkansas and Tennessee. **(F)** Arkansas and Texas.

Despite an initial surge in Louisiana, driven mostly by cases in the New Orleans metro area where COVID-19 infection grew rapidly relative to other states in the early months of the pandemic (27), Figure 1 reveals that the COVID-19 case rates in the region appear to be moving fairly synchronously.

Descriptive statistics for the 7-day moving average of cases and case infection rates is displayed in Table 1 below for the entire period as well as for the pre- and post-reopening periods. Despite Texas experiencing more COVID-19 cases overall and during each of the two partitioned time periods, the case rate is actually highest in Tennessee and Arkansas overall. Additionally, the case infection rate increased from the pre- to post-reopening period for all states (see Table 1). In particular, Missouri and Oklahoma case infection rates increased at the steepest rate, with case infection rates increasing by over 400% from pre- to post-reopening while Arkansas and Tennessee both experienced nearly 250% increases in their average COVID-19 case infection rate from the early stages of the pandemic to the time period following IHE reopening (see Table 1).

**Table 1:** Means and standard deviations by state and time period for COVID-19 case and case rate 7-day moving averages.

	**Overall** **(** ***N*** **=** **269)**		**March-July** **(** ***N*** **=** **130)**		**August-December** **(** ***N*** **=** **139)**	
**State**	**Cases**	**Case Rate**	**Cases**	**Case Rate**	**Cases**	**Case Rate**
Arkansas	698 (551)	23.2 (18.3)	308 (258)	10.2 (8.6)	1,064 (499)	35.3 (16.6)
Louisiana	1,003 (677)	21.5 (14.5)	848 (618)	18.2 (13.3)	1,149 (698)	24.7 (15.0)
Mississippi	682 (478)	22.8 (16.0)	425 (347)	14.3 (11.6)	922 (460)	30.9 (15.4)
Missouri	1,294 (1,257)	21.1 (20.5)	346 (324)	5.7 (5.3)	2,179 (1,155)	35.6 (18.9)
Oklahoma	903 (974)	22.9 (24.7)	274 (317)	7.0 (8.0)	1,490 (1,015)	37.8 (25.7)
Tennessee	1724 (1,605)	25.5 (23.7)	755 (681)	11.2 (10.1)	2,629 (1,692)	38.8 (25.0)
Texas	4,983 (3,450)	17.4 (12.0)	3,046 (3,119)	10.6 (10.9)	6,795 (2,679)	23.7 (9.3)

These descriptive findings are in line with the recent surge of COVID-19 across the United States (regardless of fall IHE plans).

### Instantaneous Synchrony

Instantaneous synchrony was assessed using linear correlation between each pairwise, bivariate time series within the region from March to December at a time lag of 0 days. Results indicated that Arkansas COVID-19 case infection rates had the greatest instantaneous synchrony with Tennessee and Missouri, with linear correlation coefficients of 0.94–0.93, respectively. However, instantaneous synchrony with Arkansas COVID-19 case infection rates was significant for all neighboring states with linear correlation coefficients between 0.70 and 0.94.

Instantaneous synchrony with Arkansas during the pre-reopening period from March to July was significant for all neighboring states with linear correlation coefficients between 0.74 and 0.93. Instantaneous synchrony with Arkansas increased for three of the six neighboring states following IHE reopening: Louisiana, Missouri, and Tennessee.

### Synchrony

While mean synchrony with Arkansas appeared to be highest for Texas, this synchrony appeared to be no more than random chance and the effect size was quite small (see Table 2). However, utilizing the surrogate synchrony algorithm to estimate the dyadic coupling between the COVID-19 processes between Arkansas and its contiguous neighbors from March to December, results indicated that significant in-phase synchrony in case infection rates is present between Arkansas and three of its contiguous neighbors: Mississippi, Missouri, and Tennessee.

**Table 2:** Overall synchrony with Arkansas (*N* = 269).

**State**	**$\bar{\text{Z}}$_**Abs**_**	**ES_**Abs**_**	***p*-Value**	**ES_**NoAbs**_**	**ES_**Lead**_**
Louisiana	0.415	−0.064	0.7062	3.235	−0.294
Mississippi	0.393	0.206	0.0402	0.223	−0.281
Missouri	0.442	0.292	0.0066	0.875	0.282
Oklahoma	0.316	−0.154	0.9047	0.951	0.334
Tennessee	0.446	0.194	0.0498	1.092	0.396
Texas	0.585	0.097	0.2048	0.633	0.165

*${\bar{Z}}_{\text{Abs}}$ indicates mean synchrony.*

*ES_Abs_ indicates effect size of synchrony.*

*ES_NoAbs_ indicates effect size of synchrony, with the ability to differentiate anti-phase and in-phase.*

*ES_Lead_ indicates effect size for directionality of entrainment*.

Missouri had the highest level of overall synchrony with Arkansas during the time period (${\bar{Z}}_{\text{Abs}}$ = 0.44, ES_Abs_ = 0.29, *p* < 0.01). Positive effect sizes when no absolute cross-correlation values are utilized in SUSY, as indicated by ES_NoAbs_, suggest in-phase synchrony. The highest levels of in-phase synchrony with Arkansas were detected in Louisiana, Tennessee, Oklahoma, and Missouri.

The effect size for the directionality of entrainment, as indicated by ES_Lead_, suggests that COVID-19 case infection rates in Arkansas somehow entrain those in Louisiana and Mississippi, whereas the rates in the remaining neighbor states entrain the process in Arkansas.

Lagged cross-correlations for the entire bivariate time series were calculated, with the highest observed lagged correlations existing between Arkansas and Tennessee at a lag of 3–6 days.

### Pre- and Post-reopening Synchrony

To further explore the potential effect of IHE reopening on the regional synchrony of COVID-19, synchrony was examined for the shorter pre- and post-reopening windows.

During the early months of the pandemic, effect sizes indicate that the magnitude of synchrony with Arkansas was greatest for Tennessee and Missouri, with Arkansas being largely entrained by the COVID-19 infection rate trends in these states (see Table 3).

**Table 3:** Comparison of March–July to August–December synchrony with Arkansas.

	**March-July (** ***N*** **=** **130)**		**August-December (** ***N*** **=** **139)**	
**State**	**ES_**Abs**_**	**ES_**Lead**_**	**ES_**Abs**_**	**ES_**Lead**_**
Louisiana	−0.161	−0.131	0.132	0.084
Mississippi	0.180	−0.580	0.190	0.279
Missouri	0.221	0.690	0.344	1.150
Oklahoma	−0.290	−0.111	0.333	0.868
Tennessee	0.252	0.858	0.417	0.499
Texas	0.011	0.222	0.241	0.285

*ES_Abs_ indicates effect size of synchrony.*

*ES_Lead_ indicates effect size for directionality of entrainment*.

Following IHE reopening and subsequent cross-border travel, moderate gains in the magnitude of in-phase synchrony with Arkansas were observed for all neighbor states. While the magnitude of synchrony was still greatest between Arkansas and two of its contiguous neighbors, Tennessee and Missouri, the directionality of entrainment effect sizes indicated that COVID-19 was predominantly in-flowing to Arkansas from all of its neighbors. The magnitude of observed entrainment for COVID-19 with Arkansas, while increasing in magnitude for all states except Tennessee, reversed direction for Oklahoma and was greatest for Missouri and Oklahoma following IHE reopening (see Table 3).

It appears that there is a spatiality to COVID-19, with certain neighboring states having case infection rates that are significantly synchronized in timing and in form with moderate-to-large effect sizes. This significant synchrony within the region is in stark contrast to the non-significant synchrony in case infection rates that was observed during the same time period between Arkansas and a non-neighboring region, such as New York city (see Supplementary Figure 1).

The synchrony of contiguous neighbors within a spatial region appears to be greatest at a lag time consistent with COVID-19 symptom onset. Using Google COVID-19 Community Mobility Reports, Arkansas movement from the pre- to post-reopening windows did increase, with a 19% increase in workplace mobility, a 26% increase in retail mobility, and a nearly 70% increase in transit mobility (28). Accordingly, this synchrony is potentially intensified as resultant cross-border movements and community mobility increase following IHE reopening.

## Discussion

Despite immense adjustments to instruction at IHE, a *New York Times* survey of nearly 2,000 IHE revealed that as of December 11, 2020, nearly 400,000 cases and almost 100 deaths were linked to such institutions (29). As noted by Walke et al., young adults contributed to large regional COVID-19 surges in the Southern United States during the summer months and, with the reopening of IHE, the virus spread would not be limited to the campus community; IHE function within their surrounding communities and virus spread within IHE necessarily poses a risk for areas where they are located (30). Following IHE reopening, case infections and deaths did increase at rates above the national average in communities with student populations of ~10% or more (12, 13).

Facing financial consequences if some level of in-person instruction was not offered, IHE implemented a variety of strategies to mitigate and prevent the spread of COVID-19 on campus during fall 2020 (30). Despite these strategies, IHE became a potential source of community spread in many parts of the United States (12, 13, 29). While transmission among students, staff, faculty, and the greater community is complex, testing proved one possible strategy to effectively minimize the spread at IHE allowing for early detection, more effectual contact tracing, and the implementation of rapid isolation and quarantine. However, previous research suggests that testing strategies at IHE should involve repeated testing and, potentially, testing that occurs every 2 days (8, 31).

As noted by Bradley et al. and, especially given the financial constraints already placed on many IHE during this time, reopening plans that require testing every 2 days are simply not feasible for all campuses (32). However, a combination of strategies and behavioral interventions could be nearly as effective. With financial consequences still more grave and an escalating winter surge of COVID-19 throughout the United States, additional planning for how IHEs might best approach instruction in 2021 to maintain financial viability while safeguarding public health of the broader community is essential.

IHE campus communities are unavoidably linked and intertwined with their surrounding communities, requiring close partnerships between the two (30). The results of the current study suggest that this inevitable interrelationship between campus communities and surrounding communities is potentially much broader in scope, with neighboring areas (e.g., contiguous states from which student populations are drawn) displaying significant COVID-19 synchrony with one another. Utilizing surrogate synchrony analysis, results suggest that a particular state could: (1) examine the synchrony over time of COVID-19 with particular neighbors, which need not be defined as contiguous states, (2) determine the directionality of entrainment, (3) analyze the impact of particular interventions and/or reopening, and (4) use such analyses to inform the development of IHE and public health policy measures to slow the spread of COVID-19. For instance, if synchrony is detected with the direction of entrainment in-flowing to a particular state or campus community, IHE testing resources could be targeted so that students first arriving on campus from particularly synchronous areas receive more repetitive testing compared to other students. Alternatively, detectable synchrony could guide decisions about the timing of IHE holiday/vacation breaks and/or whether or not to allow students to engage in cross-state or cross-region travel.

There are limitations to the current study. The CDC data are provisional in nature. Additionally, state reporting of data differed over the period from March to December. Some states reported confirmed cases and, then, began reporting both confirmed and probable cases while some states were still only reporting confirmed cases as late as December 17, 2020. Additionally, all reported data on COVID-19 cases were necessarily connected to testing availability, which may have differed between the U.S. states considered in this study at different time points during the study period. Different age groups and other individuals that might have presented as asymptomatic infections and, consequently, were less likely to be tested also suggested that the data used in this study actually underestimated the infection. Even if tested, testing in particular locations also had different turnaround times, resulting in different case reporting delays for the various states. Due to the variability of the COVID-19 cases reported daily, the data utilized in the study involved 7-day moving averages that could have inflated some cross-correlation calculations within the SUSY algorithm. Moreover, the number of observations in each time series, especially when looking at pre- and post-reopening series, are relatively short when attempting to analyze longer-term trends and synchronous properties between regional neighbors. The relatively short length of the series also limits the statistical power and restricts alteration of algorithm parameters, such as segment size or maximum time lag. Additionally, the SUSY algorithm is a measure of dyadic coupling and, thus, has been utilized multiple times in the current study to explore all pairwise, bivariate synchrony between Arkansas and its six contiguous neighboring states. Future work would look to extend the surrogate synchrony approach to more than two time series.

However, beyond extending the SUSY algorithm, future work could also involve the use of synchrony algorithms for virus surveillance across space and time, exploring the synchrony of COVID-19 or other infectious diseases between major travel hubs, such as highly connected major airports (i.e., “neighbors” in a transportation network). Future work could also look at the dyadic coupling and synchronous behavior of COVID-19 with other potentially correlated time series (e.g., weather patterns, economic measures, and mobility data,).

Keeping IHE students on campus is known to limit the mutual exposure between the campus and surrounding communities (30, 32). While many schools that implemented basic mitigation strategies (i.e., distancing, masking, hand hygiene, ventilation, and staying home when symptomatic) were able to prevent large outbreaks during fall 2020 (33), limiting secondary transmission by retaining isolated cohorts further minimized the risk of transmission. However, the politicization of the pandemic now prevents basic yet effective mitigation strategies, such as wearing a mask at some IHE, including in Arkansas (34). Additionally, shared/congregate housing is common in IHE, for example, dormitories, making isolation of cohorts and contact tracing further complicated. Therefore, without firm travel restrictions on the movement of students both in the surrounding local community and in cross-border travel, individual mobility of students is unlikely to decline to a level that would prevent the influx of COVID-19 or prevent any potential external outbreaks connected to the IHE (3). Despite limitations, this study suggests that there is a spatiality to COVID-19 that is not simply patterned on the local economy but also likely connected to the flow patterns of the IHE students within and across regions (7). Moreover, this study demonstrates how SUSY can be utilized to detect regional COVID-19 synchrony, to determine the direction of entrainment, to evaluate intervention effects on regional synchrony, and to guide targeted IHE and public health policy.

## Data Availability Statement

Publicly available datasets were analyzed in this study. This data can be found here: COVID-19 Case Surveillance Public Use Data is accessible through the CDC COVID Data Tracker: https://covid.cdc.gov/covid-data-tracker.

## Author Contributions

The author confirms being the sole contributor of this work and has approved it for publication.

## Conflict of Interest

The author declares that the research was conducted in the absence of any commercial or financial relationships that could be construed as a potential conflict of interest.

## Publisher's Note

All claims expressed in this article are solely those of the authors and do not necessarily represent those of their affiliated organizations, or those of the publisher, the editors and the reviewers. Any product that may be evaluated in this article, or claim that may be made by its manufacturer, is not guaranteed or endorsed by the publisher.
