# Supplementary figures and images for: Regional COVID-19 Dynamics: Surrogate Synchrony in Case Infection Rates
Source: Front Public Health. 2021 Aug 26;9:647441. doi: 10.3389/fpubh.2021.647441 (PMC8426435; doi:10.3389/fpubh.2021.647441)

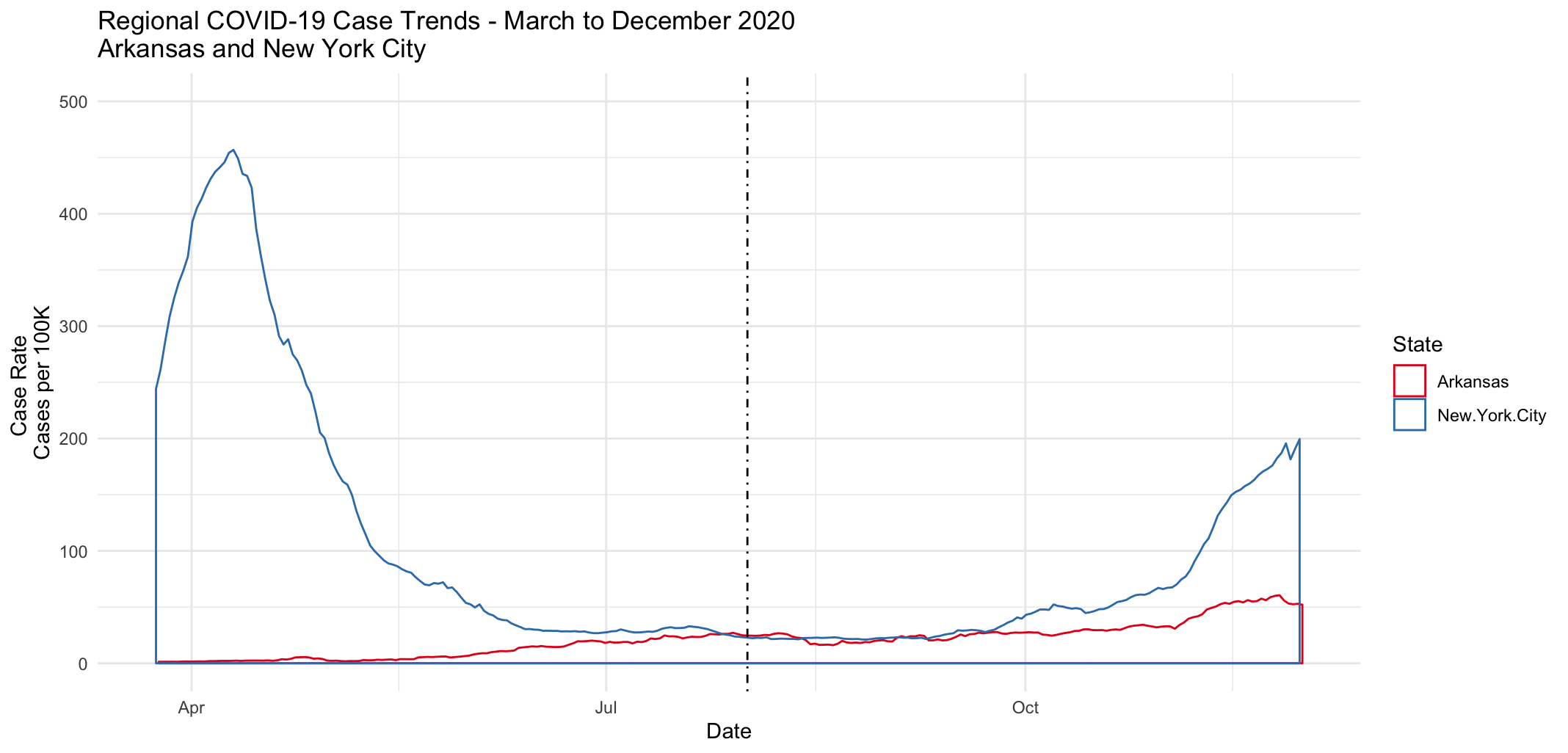

Supplement: Supplementary file 1 [file Image_1.JPEG]
